# Nutritional distinction of Bolivian Quinoa Real compared to global varieties

**DOI:** 10.1038/s41538-026-00735-5

**Published:** 2026-02-02

**Authors:** J. Mauricio Peñarrieta, Erick Loayza, Javier A. Linares-Pastén

**Affiliations:** 1 Instituto de Investigaciones Químicas IIQ, Universidad Mayor de San Andrés UMSA, Av. Villazón N° 1995, 0201-0220, La Paz, Bolivia; 2 Unidad de Ecología Acuática, Instituto de Ecología, Facultad de Ciencias Puras y Naturales, Universidad Mayor de San Andrés, La Paz, Bolivia; 3 Department of Veterinary and Biosciences, Faculty of Veterinary Medicine, Ghent University, Merelbeke, Belgium; 4 Biotechnology and Applied Microbiology, Department of Process and Life Sciences Engineering. Faculty of Engineering LTH, Lund University, Lund, Sweden

**Keywords:** Biochemistry, Plant sciences

## Abstract

Quinoa is globally recognised for its nutritional value, and its production has recently expanded worldwide. However, Quinoa Real (Royal Quinoa), a landrace group grown exclusively in the *Intersalar* zone of Bolivia, stands out for its grain quality and adaptation to extreme environments, such as high altitude, high salinity, intense UV radiation, aridity, and temperature fluctuations. This study compares the nutritional composition of 13 well-established commercial quinoa samples from 9 countries, including Quinoa Real white, red, and black. Analyses covered granulometry, proximate composition, fatty acid and amino acid profiles, vitamins, and minerals. Multivariate analyses (PCA and nMDS) revealed clear compositional distinctions for Quinoa Real, including higher levels of dietary fibre, ash, phytosterols, and essential minerals. It also exhibits a more favourable fatty-acid profile, higher levels of several vitamins, and a well-balanced essential amino acid profile. These results show that Quinoa Real is not only a nutritional outlier but also a valuable agrobiodiversity resource with implications for food security, functional foods, and sustainable production in the face of global dietary and environmental challenges.

## Introduction

Quinoa (*Chenopodium quinoa*) is not a cereal, but rather a member of the family Amaranthaceae, specifically the subfamily Chenopodiaceae. It is an ancient crop that was domesticated approximately 5,000 years ago at an elevation of 3,800 m (m.a.s.l.) in the surrounding regions of Lake Titicaca, which is shared by Bolivia and Peru^1^. Indeed, archaeological studies indicate that quinoa, along with potatoes (*Solanum tuberosum*) and llamas (*Lama glama*), formed a stable food source for Tiwanaku civilisation^2^. It was one of the most important pre-Hispanic spiritual and political centres in South America, extending mainly to territories that are now Bolivia, Peru, and Ecuador^3,4^. Moreover, since quinoa domestication, several historical events have made it one of the most attractive foods today and promoted its worldwide expansion (Table 1).

**Table 1:** The history of quinoa

Period/Date	Event	Reference
Prehistoric		
~5000 BCE	**Domestication. According to archaeological studies**. Quinoa was domesticated in the surroundings of Lake Titicaca. in today’s Bolivia and Peru.	^1,2^
Ancient		
~2000 BCE - 1000 CE	**Tiwanaku Civilisation**: Quinoa was considered one of the staple crops that sustained the diet and agriculture of the Tiwanaku civilisation around Lake Titicaca.	^2^
~1200 CE - 1532 CE	**Inca Empire**: Quinoa became one of the primary food sources for the Incas. Revered as a sacred crop. it was called “chisaya mama” or “mother of all grains.”	^60,61^
Colonial		
1532 CE	**Hispanic Conquest:** European crops. such as wheat and barley. substituted for quinoa. decreasing its production.	^62,63^
20th Century		
1970s	**Scientific Interest**: Quinoa’s nutritional value is beginning to be recognised.	
The late 1980s	**International Awareness**: Quinoa’s nutritional benefits started gaining attention in North America and Europe.	
1993	**Space Suitability:** NASA has considered quinoa a suitable candidate for space missions due to its ease of cultivation and adaptability to controlled environments.	^64^
21st Century		
The early 2000s	**Health Food Trend**: Quinoa has become popular globally as a health food due to its gluten-free status and high protein. fibre. and essential amino acid content.	
2013	**International Year of Quinoa**: The United Nations declares 2013 as the International Year of Quinoa to recognise its cultural significance and potential for food security.	
2017	**First high-quality genome sequence:** The genome of the coastal Chilean quinoa was sequenced and assembled. BioSample accession code: SAMN04338310.	^65^
2020 s	**Global Expansion**: Quinoa is now grown in North America. Europe. Asia. and Africa. Varieties are expanding. and production is increasing.	^20,66^

From its domestication to its global expansion today.

Quinoa has drawn attention in recent decades due to its high nutritional value, resistance to climate change, and adaptability to growing in poor soils^4^. On the other hand, quinoa is a gluten-free crop^5,6^. It contains a high level of fatty acids, vitamins, dietary fibre, and a high concentration of essential amino acids^7–10^. It is often referred to as a “superfood”^11,12^.

One of the nutritional components that makes quinoa attractive is its protein content. Although quinoa contains significantly lower levels of protein than common legumes, it has all nine essential amino acids, which is not usual among plant proteins. Legumes (Fabaceae) are the most protein-rich food plants. For example, peas (23%), fava beans (27%), lupines (32%), and soybeans (38%)^13^. In contrast, quinoa is not a legume, but it is particularly rich in methionine, lysine, and threonine, which are often deficient in cereals and legumes^14,15^. Indeed, quinoa is sometimes confused with a cereal (Poaceae) or referred to as a “pseudocereal”. The correct way to refer to quinoa should be amaranthaceous. Quinoa has higher protein levels than the most common cereals, ranging from 8% to 22%, depending on variety and growing conditions^16,17^. As a reference, common cereals such as wheat (10–15%), rice (7–8%), and corn (9–14%) have lower protein content^14,18,19^.

**Fig. 1:**
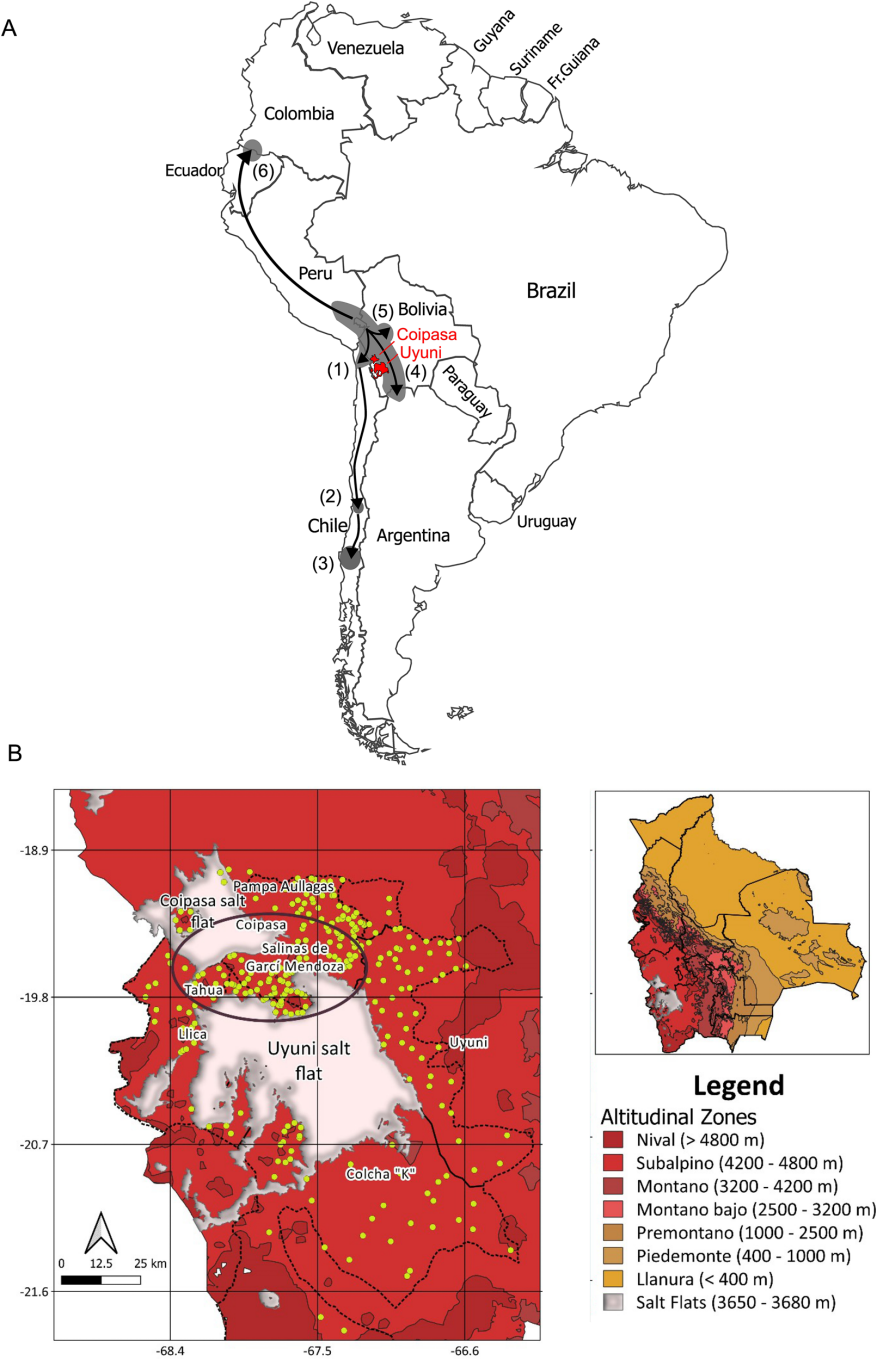
Quinoa ecotype distribution in the Andes. **A** Biodiversity dynamics of quinoa including six ecotypes (Modified from Fuentes et. al 2012^67^), (1) Salares (northern Chile – southern Bolivia), (2) Lowland/coastal (central Chile), (3) Lowland/coastal (southern Chile), (4) Highlands (Perú –Bolivia – Argentina), (5) Yungas (Bolivia), and (6) Inter-Andean Valley (Ecuador – Colombia). **B** The ecotype Salares includes quinoa real varieties, distributed in the surrounding of the Coipasa and Uyuni salt flats.

Quinoa has diversified into a wide range of varieties over time. In the Andean region, it has been distributed at altitudes from sea level to 4000 m. They have expanded north and south of Lake Titicaca, reaching Colombia to the north and Chile to the south (Fig. 1A). Thus, the following ecotypes (typologies) that differ in morphology, phenology, resistance to biotic and abiotic factors, and others were described^1^: (1) Altiplano, cultivated in high altitudes ( > 3,500 m) in the proximities of Titicaca Lake and northern highlands (Bolivia and Peru), it has large seeds and it is cold-tolerant; (2) Valley (Inter-Andean Valleys), growth in the central Andes (Bolivia, Peru, Ecuador and Colombia), between 2000 and 3500 m, and the seed size is intermediate; (3) Salares, found in the surroundings of the salt flats Uyuni and Coipasa (Bolivia), between 3600 and 4200 m, tolerate high salinity and drought, their seeds are large; (4) Coastal, grown in the Chilean coastal zones from 0 to 500 m, and are adapted to maritime clime; (5) Yungas (Subtropical), cultivated in the Eastern Andean slopes of Yungas region in Bolivia, in a humid clime, low-altitude, between 500 and 2,000 m, they are adapted to the humidity and have short cycles. In recent years, quinoa has expanded to North America, Europe, Africa, Asia, and Oceania^20^.

Bolivia and Peru are still the world’s largest quinoa producers, with approximately 113,376 and 44,707 tons per year, respectively^21,22^. Both countries account for approximately 80% of global production, while Ecuador (883 t/y, FAOSTAT, 2022) is the third-largest producer. Colombia has recently increased its quinoa production (375 t/y)^23^. Other countries that are starting to commercialise quinoa include the USA, Canada, Spain, China, and India^24,25^. While new countries entered the quinoa market, Bolivia dramatically reduced its production, from 71,000 tons per year in 2018 to 42,700 tons per year in 2022 (FAOSTAT, 2022). The steadily growing global quinoa market has socioeconomic and environmental impacts on producers in the Andean countries. The debate on mitigating the negative environmental impact on Andean-producing countries should consider the question of ownership regarding quinoa from Andean nations as a strategy to create fair access to the global market through sustainable production^19^.

Although some quinoa varieties have spread around the world, Quinoa Real grows only in Bolivia. This unique variety belongs to the Salar ecotype, which encompasses 54 native cultivars found around the Uyuni and Coipasa salt flats (Fig. 1B)^26–28^. This region, known as the *Intersalar* zone, is characterised by its extreme environmental conditions. It is located at a high altitude, ranging from 3,800 to 4,800 m, and is highly exposed to UV radiation. The climate is arid, with low annual rainfall, between 150 and 200 mm. The temperature fluctuates drastically, from very low at night to high during the day. The soils are saline and mineral-rich but deficient in nitrogen^29,30^. These extreme conditions make Quinoa Real unique and open the door to research how these factors might affect its nutritional profile. Geographically, this zone is between the Oruro and Potosí departments and includes production centres such as Lica, Salinas de Garci Mendoza, and Pampa Aullagas^26,30^.

To differentiate Quinoa Real in the global market, Bolivian producers, processors, exporters, and public and private institutions have hypothesised that this variety has unique nutritional attributes compared to other quinoa varieties. However, while the morphological differences of Quinoa Real, such as its size, thickness, and colour, are evident, there is no conclusive evidence to confirm its superior nutritional properties (Quinoa Sector Strategy, Bolivian Ministry of Rural Development and Land, Bolivia, 2017)^31^.

The present study aims to describe the nutritional content of Quinoa Real, distinguishing it from varieties from other countries.

## Results

**Fig. 2:**
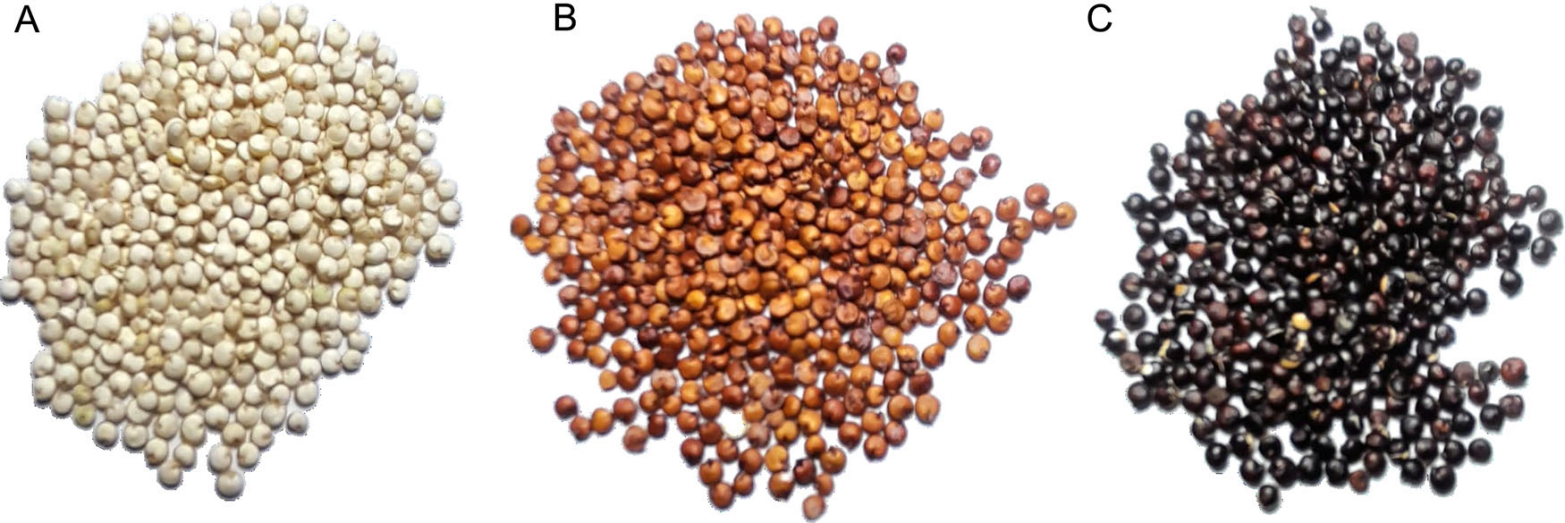
Representative varieties of Quinoa Real were subjected to the present study. **A** Quinoa Real white. **B** Quinoa Real red. **C** Quinoa Real black.

The primary characteristics of Quinoa Real are the morphological differences, particularly its large seeds (greater than 1.8 mm), thickness, colour, and appearance. The grains can be white (Real Blanca, Rosa Blanca, etc.), red (Pandela, Toledo, etc.), and black (Pisankalla) (Fig. 2)^32^. These cultivars were obtained through mass selection, and their conservation was achieved decades ago through varietal purification by producers^32^. The local names are: Real Blanca, Pandela, Toledo, Otusaya, Qanchis Blanco, Maniqueña, Pisanqalla (red), Cha’aku, Qíllu Rosa Blanca, Achachino, and Chu’llpi. Among them, Qanchis Blanco and Maniqueña, early and semi-precocious cultivars, are highly adaptable to climate change^32^.

### Granulometry

**Fig. 3:**
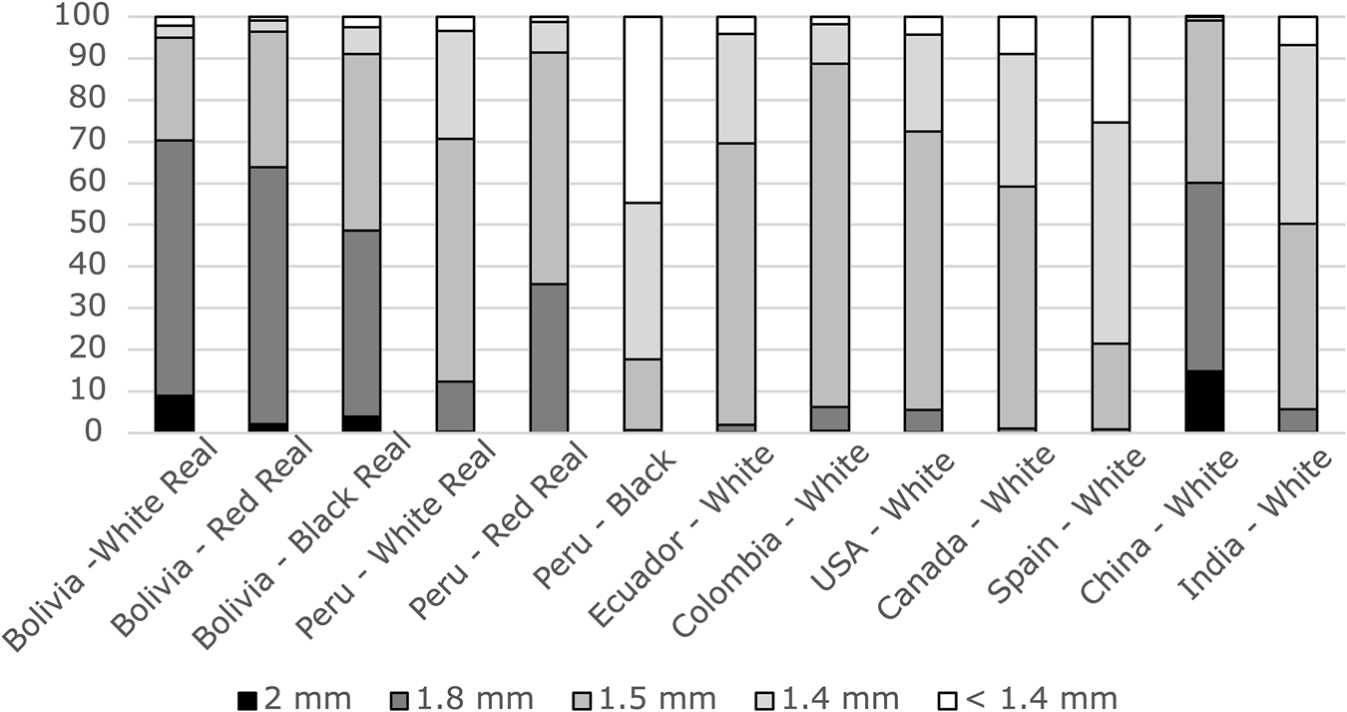
Granulometric analysis of varieties of quinoa. Bar plots show the particle-size distribution for each quinoa variety analysed.

Seed size analysis was conducted using various sieves of 2.2, 1.8, 1.5, and 1.4 mm, as shown in Fig. 3. The results indicated that Quinoas Real from Bolivia had larger seeds than other samples, particularly at the 1.8 mm and 2.2 mm sieve sizes, compared with the grain size standard for quinoa in the Codex Alimentarius^33^. It is worth noting that the quinoa sample from China had 14% large seeds at 2.2 mm, slightly higher than the 9% observed in Bolivian Quinoa Real white. However, the percentage of large seeds in the Bolivian sample was higher at 1.8 mm than in the Chinese quinoa.

### Proximate Composition analysis: protein, fat, carbohydrate, ash, and moisture

The chemical composition of quinoa samples collected from various countries is summarised in Table 2. Bolivian Quinoa Real (white, red, and black) showed markedly higher levels of dietary fibre and ash. compared to quinoa from other countries (Table 2). Red Real from Bolivia showed a significantly elevated fibre content and phytosterol. White quinoa from Canada also showed a high level of phytosterol, but lower than the red Real from Bolivia.

**Table 2:** Chemical analysis of quinoa varieties from different countries

Quinoa varieties	Protein (g/100 g)	Fat (g/100 g)	Saturated fatty acids (g/100 g)	Carbohydrates (g/100 g)	Total sugars (g/100 g)	Phytosterol (mg/100 g)	Total dietary fibre (g/100 g)	Salt ^(1)^ (g/100 g)	Ash (g/100 g)	Dry matter (g/100 g)	Energy (KJ/100 g)	Energy (kcal/100 g)
**Bolivia**												
White Real	11.3	7.3	1.1	32.9	< 0.5*	< 1*	36.3	0.02	2.9	90.7	1311.9	315.1
Red Real	10.9	3.3	0.5	10	< 0.5*	2.2	62.7	0.01	3.6	90.5	979	238.7
Black Real	9.4	2.6	0.4	55.8	3.9	< 1*	18.7	0.02	3.6	90.1	1354.2	321.6
**Peru**												
White Real	13.1	5.9	0.8	48.4	0.9	< 1*	19.5	0	2.7	89.6	1419.8	338.1
Red Real	9.3	7.2	1	54.6	4.2	< 1*	16.1	0	2.6	89.8	1481.5	352.6
Black	11	3.6	0.5	53.7	3.6	< 1*	15.9	0.02	2.6	86.8	1360.3	323
**Ecuador**												
White	14.4	7.5	1	50.9	< 0.5*	< 1*	13	0	2.5	88.3	1491.6	354.7
**Colombia**												
White	14.6	6.6	0.7	52.8	1.2	< 1*	9.8	0.01	2.3	86.1	1468.4	348.6
**USA**												
White	10.3	6.7	0.8	64.1	3.9	< 1*	6	0	2.2	89.3	1560.7	369.9
**Canada**												
White	9.6	6.7	1	55.6	1.3	1	16.8	0	2.3	91	1490.7	354.7
**Spain**												
White	10.5	5.6	0.8	64.5	0.8	< 1*	8.2	0.01	2.4	91.2	1547.8	366.8
**China**												
White	12.5	5.1	0.6	59.9	3.9	< 1*	8.5	0	2.2	88.2	1487.5	352.5
**India**												
White	15.4	3.6	0.5	62.6	<0.5*	< 1*	7.1	0.03	1.9	90.6	1516	358.6

(1) Calculated from NaCl.

*Below the quantification level.

Dietary fibre is associated with improved gastrointestinal function, better glycaemic regulation, and reduced serum cholesterol, among other beneficial effects^34^. Phytosterols are associated with anti-inflammatory effects and cholesterol-lowering activity, which may contribute to cardiovascular health^35,36^. Thus, the compositional traits highlight the distinct functional potential of Bolivian Quinoa Real compared to other quinoa varieties evaluated.

White quinoa from India exhibited the highest protein content, whereas Peru samples showed elevated carbohydrate and sugar levels. Notably, the Bolivian samples showed a comparatively lower protein content, a characteristic attributed to the specific soil conditions in the *Intersalar* zone^30^. In this line, we recently found that Quinoa Real varieties from Bolivia presented slightly lower protein levels than the tropicalized variety, which is adapted to grow in the lowlands of Bolivia^37^.

**Fig. 4:**
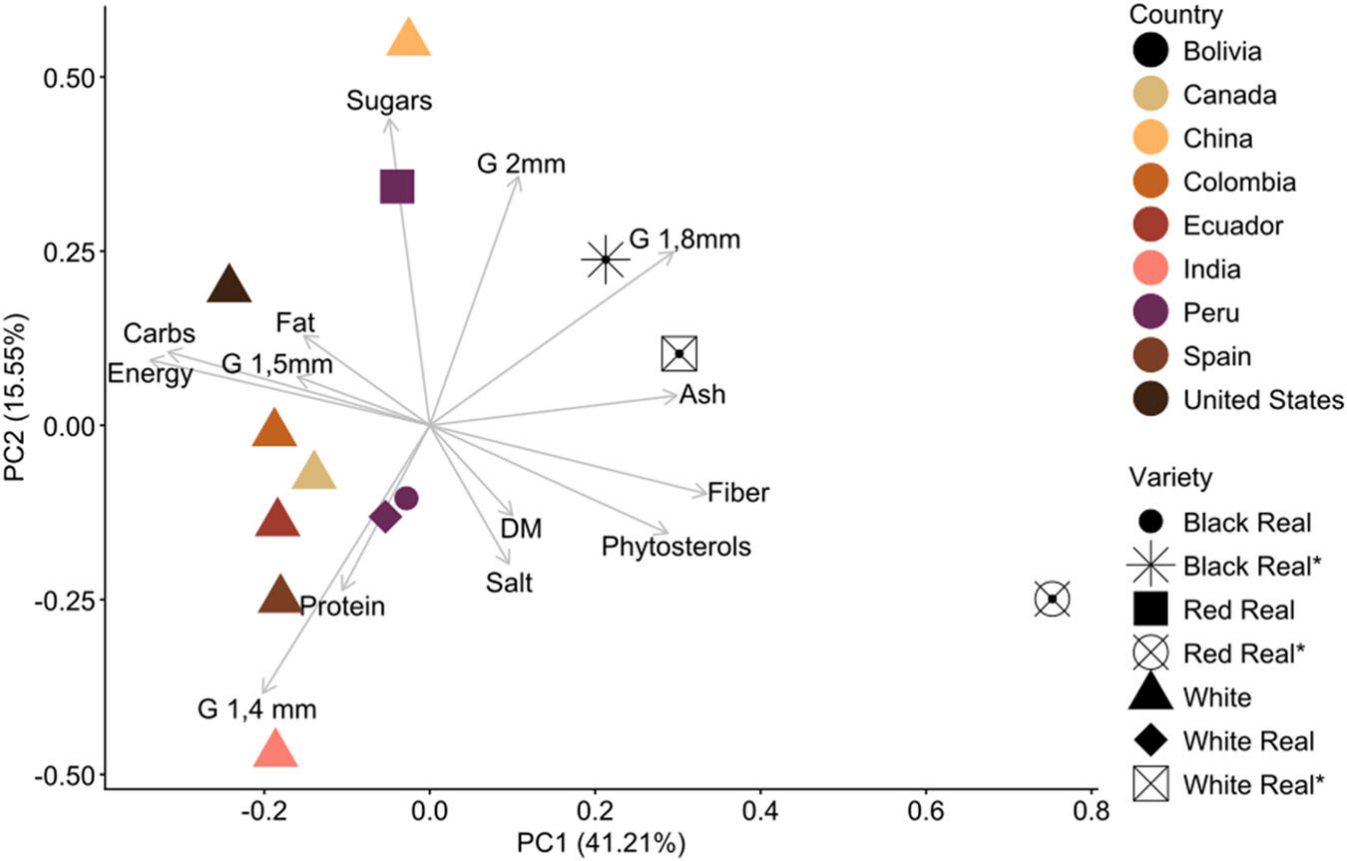
Principal Components Analysis (PCA) biplot showing variations in the nutritional composition and grain size parameters of 13 quinoa samples from different countries. The combined effects of PC1 and PC2 explained 57% of the total variance.

The PCA analysis is shown in Fig. 4. The first two principal components accounted for 56.8% of the total variance, with PC1 accounting for 41.2% and PC2 accounting for 15.6%. PC1 was primarily structured by opposing contributions from energy-rich constituents and ash- and fibre-associated traits. High negative loadings for carbohydrates and energy, along with moderate negative contributions from fat and sugars, suggest that samples with greater concentrations of macronutrients contributing to caloric density tended to cluster on the negative end of this axis. Conversely, positive loadings for fibre, ash, and phytosterols position these traits at the opposite end of the gradient. Thus, PC1 reflects a nutrient-density vs. structural/mineral composition trade-off, distinguishing samples richer in digestible energy from those characterised by higher mineral and dietary fibre content. PC2, in contrast, was structured mainly by strong positive contributions from sugars and granulometry at 2 mm, coupled with moderate positive contributions from granulometry at 1.8 mm and fat. These patterns indicate that PC2 captures a gradient related to seed particle size and soluble carbohydrate content, with samples with larger grain fractions exhibiting higher sugar levels. Negative loadings for protein, salt, and ash suggest that samples positioned at the lower end of PC2 were comparatively richer in structural or mineral-associated components rather than soluble carbohydrates. In summary, PC1 and PC2 showed a nutritional variability, where we could observe (1) a caloric/structural nutrient contrast driven by macronutrient composition, and (2) a granulometric–carbohydrate gradient reflecting physical seed characteristics and sugar content.

### Amino acid profile

**Fig. 5:**
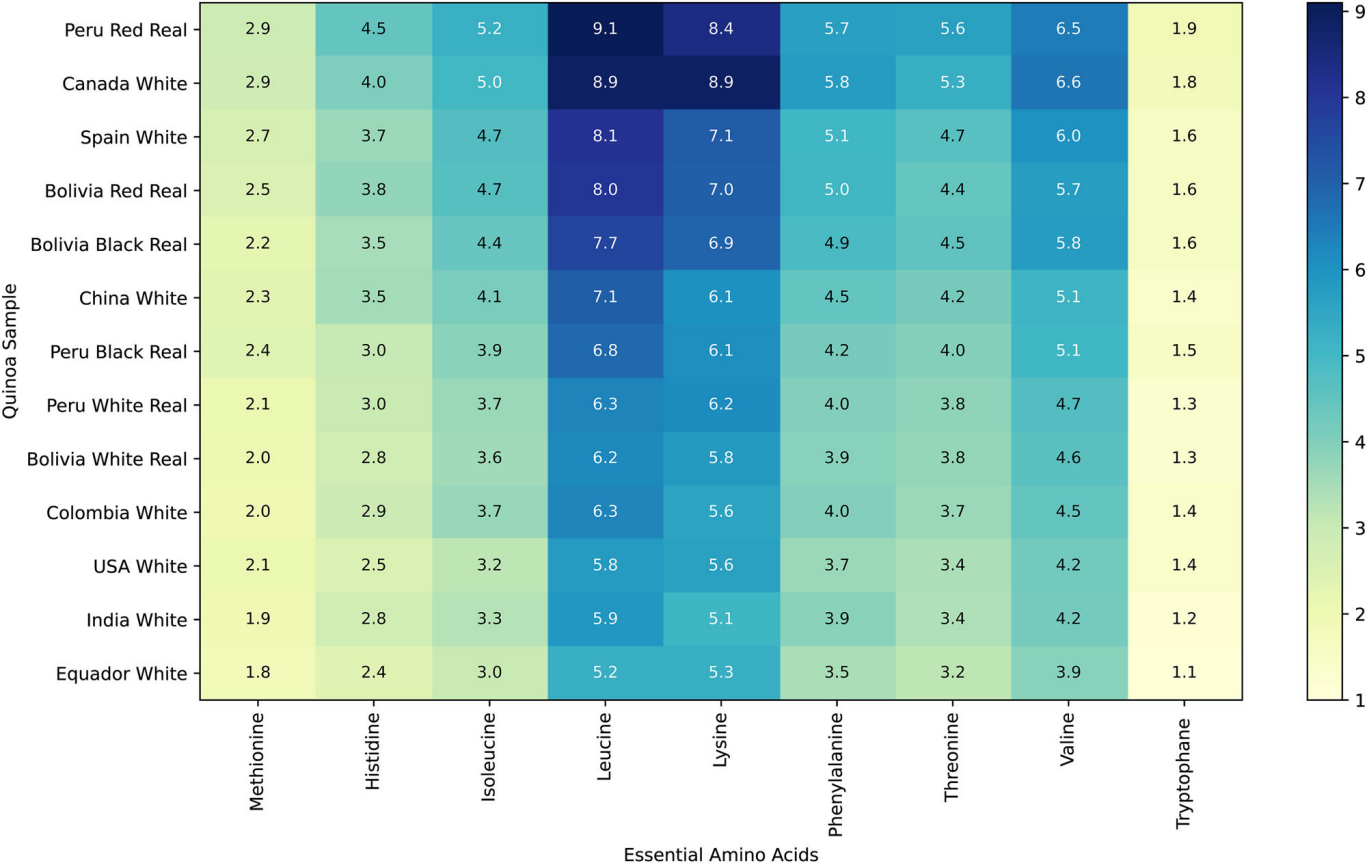
Heatmap of essential amino acids in quinoa samples (g/100 g protein). Darker shades indicated higher concentration.

The amino acid profile is shown in Table 3, expressed as g/100 g of protein, as it indicates the quality of the protein source. All quinoa samples contain the nine essential amino acids required for human nutrition (Table 3). This makes quinoa nutritionally superior to most cereals, which lack one or more essential amino acids. The results are relatively homogeneous. The essential amino acids in their order of appearance are leucine, lysine, valine, phenylalanine, threonine, isoleucine, histidine, methionine, and tryptophan. It is worth noting that the samples from Ecuador and Canada contain similar amounts of leucine and lysine. At the same time, Spain and Colombia quinoa presented the same values for isoleucine and threonine. These results showed a similar order to that reported in the literature^38^, but phenylalanine and threonine were at lower concentrations than isoleucine. The top 5 samples by total essential amino acid content are red Real from Peru (49.8 g/100 g), Canada white (48. 6 g/100 g protein), Spain white (45.6 g/100 g protein), Bolivia Real red (44.1/100 g protein), and Real black from Bolivia (42.2 g/100 g protein) (Fig. 5).

**Table 3:** Amino acid profile (g/100 g protein) of the 13 quinoa varieties from different countries

Components	Bolivia				Peru			Ecuador	Colombia	USA	Canada	Spain	China	India
	White Real	Red Real	Black Real	White Real		Red Real	Black	White	White	White	White	White	White	White
Cystein + Cystine	1.8	2.2	2.2	1.7		2.5	1.9	1.4	1.6	1.3	2.7	2.2	1.9	1.7
Methionine	2.0	2.5	2.2	2.1		2.9	2.4	1.8	2.0	2.1	2.9	2.7	2.3	2.0
Alanine	4.4	5.3	5.3	4.4		6.3	4.7	3.8	4.4	4.1	6.4	5.6	4.7	3.9
Arginine	8.2	11.1	10.2	8.1		12.5	8.8	6.8	9.1	7.0	12.3	11.4	10.2	8.6
Aspartic acid	8.4	10.7	10.5	8.3		12.2	8.9	6.9	8.3	7.4	11.6	11.0	9.7	7.9
Glutamic acid	13.6	18.1	16.6	13.7		20.5	14.5	11.3	14.3	12.2	20.5	19.8	17.0	14.8
Glycine	5.4	6.6	6.4	5.4		7.9	5.9	4.7	6.0	5.3	7.8	7.5	5.9	5.2
Histidine	2.8	3.8	3.5	3.0		4.5	3.0	2.4	2.9	2.5	4.1	3.7	3.5	2.8
Hydroxyproline	< 0.5*	< 0.5*	0.5	< 0.5*		0.5	0.5	< 0.5*	< 0.5*	< 0.5*	< 0.5*	1.9	< 0.5*	1.3
Isoleucine	3.6	4.7	4.4	3.7		5.2	3.9	3.0	3.7	3.2	5.0	4.7	4.1	3.3
Leucine	6.2	8.0	7.6	6.3		9.1	6.8	5.3	6.3	5.8	8.9	8.1	7.1	5.9
Lysine	5.8	7.0	6.9	6.2		8.4	6.1	5.3	5.6	5.6	8.9	7.1	6.1	5.1
Ornithine	< 0.5*	< 0.5*	< 0.5*	< 0.5*		< 0.5*	< 0.5*	< 0.5*	< 0.5*	< 0.5*	< 0.5*	< 0.5*	< 0.5*	< 0.5*
Phenylalanine	3.9	5.0	4.9	4.0		5.7	4.1	3.5	4.0	3.7	5.8	5.1	4.5	3.9
Proline	3.4	5.0	4.0	3.8		5.2	4.1	3.2	3.5	3.3	5.3	5.1	4.0	3.6
Serine	4.7	5.5	5.3	4.4		6.1	4.6	3.6	4.4	3.9	6.0	5.9	5.1	4.3
Threonine	3.8	4.4	4.5	3.8		5.6	4.0	3.2	3.7	3.4	5.3	4.7	4.2	3.4
Tyrosine	2.9	3.7	3.7	3.0		4.0	3.0	2.7	2.9	2.7	4.1	3.8	3.2	2.8
Valine	4.6	5.7	5.8	4.7		6.5	5.1	3.9	4.5	4.2	6.6	6.0	5.1	4.2
Tryptophane	1.3	1.6	1.6	1.3		1.9	1.5	1.1	1.4	1.4	1.8	1.6	1.4	1.2

*Below the quantification level.

All samples analysed in this study met the nutritional requirements for adults and children, except for leucine. The white Quinoa Real from Bolivia, white Real from Peru, and white from Colombia, the USA, India, and Ecuador were found to be slightly below the limit for children’s requirements^38^^–^^40^.

**Fig. 6:**
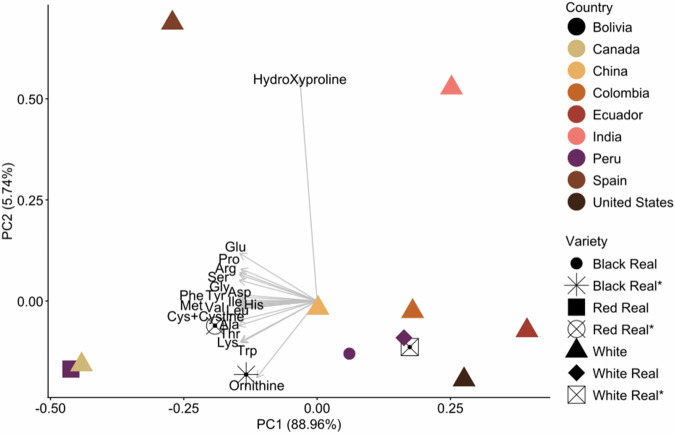
Principal Component Analysis (PCA) biplot illustrating the relationship between quinoa samples and their amino acid profiles. The biplot illustrates the relationships among quinoa samples and their amino acid compositions. Arrows represent individual amino acids contributing to sample variation.

To investigate differences among the samples, principal component analysis was used to identify patterns in amino acid composition across all samples. Fig. 6 shows the biplot of all samples with their corresponding amino acid parameters. PC1 and PC2 together explained 94% of the total variance. Correlations were observed between samples from different countries. For example, only two samples (from India and Spain) were described in terms of their hydroxyproline content, while the others were not. PC1 explained 88.9% of the variance. It was characterised by strongly negative loadings across all amino acids, including essential (e.g., leucine, isoleucine, lysine) and non-essential (e.g., glutamate, aspartate, serine) residues. This pattern suggests that all amino acids tend to vary together in the same direction, indicating a general protein content gradient rather than amino acid-specific differences. In practical terms, varieties at one end of PC1 have higher overall amino acid levels, while those at the other end have lower levels. The dominance of PC1 indicates that the main variation among quinoa varieties mainly reflects total protein content rather than differences in amino acid types. PC2 (5.7%) was primarily influenced by a strong positive loading for hydroxyproline and a large positive contribution from ornithine, while most other amino acids showed weak loadings on this axis.

Because of the extremely high multicollinearity among amino acids, with all residues loading strongly and uniformly on PC1, the PCA mainly reflects total protein abundance rather than the independent variation of individual amino acids. PC1 captures the overall nutritional potential of quinoa varieties in terms of amino acid content. Meanwhile, PC2, which explains a smaller proportion of variance, highlights secondary biochemical features, including hydroxyproline linked to structural proteins and ornithine associated with nitrogen metabolism. Therefore, these components are best interpreted as general axes of protein quantity and secondary metabolic traits, rather than as differences among individual amino acids.

Regarding the samples from Bolivia, red and black Real accounted for the majority of the amino acid content. In contrast, white Quinoa Real from Bolivia showed lower values and was more similar to White Real quinoa from Peru. The highest-amino-acid-content samples were red Quinoa Real from Peru and white quinoa from Canada, followed by red and black Quinoa Real from Bolivia, indicating superior protein quality.

### Fatty acid profile

Quinoa´s acid profile is notable for its high content of essential polyunsaturated fatty acids (PUFAs) and a nutritionally favourable ω-6 to ω-3 ratio (Table 4). These results are well in line with the literature^41^; quinoa contains the most abundant fatty acid, linoleic acid (C8:2), followed by oleic acid (C18:1) and palmitic acid (C16:0)^41,42^. PUFAs account for more than 50% of the total fatty acid composition, indicating a high-quality fat profile from a nutritional perspective, as they have several positive effects on cardiovascular disease^43,44^.

**Table 4:** Fatty acids from 13 quinoa samples collected in different countries (%)

Components	Bolivia			Peru			Ecuador	Colombia	USA	Canada	España	China	India
	White Real	Red Real	Black Quinoa	White Real	Red Real	Black	White	White	White	White	White	White	White
C4:0 butyric acid	0.16	0.14	0.12	< 0.1*	< 0.1*	< 0.1*	< 0.1*	< 0.1*	< 0.1*	0.12	< 0.1*	< 0.1*	< 0.1*
C6:0 caprolic acid	0.12	0.11	< 0.1*	< 0.1*	< 0.1*	< 0.1*	< 0.1*	< 0.1*	< 0.1*	< 0.1 *	< 0.1 *	< 0.1*	< 0.1*
C8:0 caprylic acid	< 0.1*	< 0.1*	< 0.1*	< 0.1*	< 0.1*	< 0.1*	< 0.1*	< 0.1*	< 0.1*	< 0.1*	< 0.1*	< 0.1*	< 0.1*
C10:0 capric acid	0.19	0.19	0.16	< 0.1*	< 0.1 *	< 0.1 *	< 0.1*	< 0.1 *	< 0.1*	0.13	< 0.1*	< 0.1*	< 0.1*
C12:0 lauric acid	0.28	0.28	0.25	< 0.1*	< 0.1*	< 0.1*	< 0.1*	< 0.1*	0.44	0.19	< 0.1*	< 0.1*	< 0.1*
C14:0 myristic acid	0.9	0.84	0.77	0.24	0.25	0.22	0.24	0.18	0.41	0.72	0.26	0.3	0.29
C14:1 myristoleic acid	< 0.1*	< 0.1*	< 0.1*	< 0.1*	< 0.1*	< 0.1*	< 0.1*	< 0.1*	< 0.1*	< 0.1*	< 0.1*	< 0.1*	< 0.1 *
C15:0 pentadecanoic acid	0.13	0.14	0.13	< 0.1*	< 0.1*	< 0.1*	< 0.1*	<0.1*	< 0.1*	0.11	< 0.1*	< 0.1*	< 0.1*
C16:0 palmitic acid	10.8	11.1	11.3	10.4	10.9	10.9	10.8	8.8	9.1	10.8	10.6	10.2	10.1
C16:1 palmitoleic acid	0.17	0.16	0.18	< 0.1 *	0.21	0.25	0.22	0.18	0.16	0.17	0.23	0.21	0.15
C17:0 margaric acid	< 0.1*	< 0.1*	< 0.1*	< 0.1*	< 0.1*	< 0.1*	< 0.1*	< 0.1*	< 0.1*	< 0.1*	< 0.1*	< 0.1*	< 0.1*
C17:1 heptacenoic acid	< 0.1*	< 0.1*	< 0.1*	< 0.1*	< 0.1*	< 0.1*	< 0.1*	< 0.1*	< 0.1*	< 0.1*	< 0.1*	< 0.1*	< 0.1*
C18:0 stearic acid	1.6	1.5	1.7	0.9	0.9	1.1	0.9	0.6	0.5	1.2	1	0.8	1.3
C18:1 trans elaidic acid	0.19	0.18	0.48	< 0.1*	< 0.1*	< 0.1*	0.28	< 0.1*	< 0.1*	0.15	0.15	< 0.1*	0.11
C18:1 oleic acid	26.9	28.7	24	26.9	29.1	24.6	24.6	22.2	22.6	21.1	21	23.7	21.6
C18:2 trans	0.44	0.46	0.66	0.39	0.39	0.51	0.58	0.37	0.34	0.55	1.1	0.43	0.61
C18:2 linoleic acid	43.5	42.9	46.1	47.7	45.8	52.5	53.8	59.1	53.4	50.3	55.3	51.8	55.6
C18:3 trans	< 0.1*	< 0.1*	< 0.1*	< 0.1*	< 0.1*	< 0.1*	< 0.1*	< 0.1*	< 0.1*	< 0.1*	0.15	< 0.1*	< 0.1*
C18:3 linoleic acid	7.8	6.3	7.8	5.6	7.3	3.2	3.6	3.6	6.7	7.9	4.6	8	5.7
C20:0 arachidic acid	0.47	0.55	0.46	0.52	0.5	0.5	0.43	0.45	0.34	0.38	0.47	0.42	0.37
C20:1 gadoleic acid	1.4	1.4	1.3	1.5	1.4	1.4	1.5	1.2	1.2	1.4	1.5	1.3	1.4
C20:2 eicosadienoic acid	0.11	0.11	0.12	0.14	0.11	0.15	0.15	0.14	0.12	0.15	0.17	0.11	0.15
C20:4n6 arachidonic acid	< 0.1*	< 0.1*	0.1	< 0.1*	< 0.1*	1.3	< 0.1*	< 0.1*	1.6	< 0.1*	< 0.1*	< 0.1*	< 0.1*
C22:0 behenic acid	0.65	0.76	0.71	0.71	0.6	0.67	0.68	0.7	0.5	0.56	0.77	0.59	0.61
C22:1 erucic acid	1.3	1.2	1.2	1.4	1.4	1.5	1.4	1.2	1.5	1.3	1.7	1.3	1.4
C24:0 lignoceric acid	0.27	0.44	0.42	0.31	0.29	0.32	0.25	0.34	0.26	0.28	0.39	0.34	0.31
Total Saturated Fatty Acids	15.6	16	16	13.1	13.4	13.7	13.3	11.1	11.5	14.5	13.5	12.6	13
Total Monounsaturated Fatty Acids	30	31.6	27.2	29.8	32.1	27.8	28	24.8	25.5	24.1	24.6	26.5	24.7
Total Polyunsaturated Fatty Acids	51.9	49.8	54.8	53.8	53.6	57.7	58.1	63.2	62.2	58.9	61.3	60.3	62.1
Total Trans Fatty Acids	0.63	0.64	1.1	0.39	0.39	0.51	0.86	0.37	0.34	0.7	1.4	0.43	0.72

*Below indicated quantification level.

**Fig. 7:**
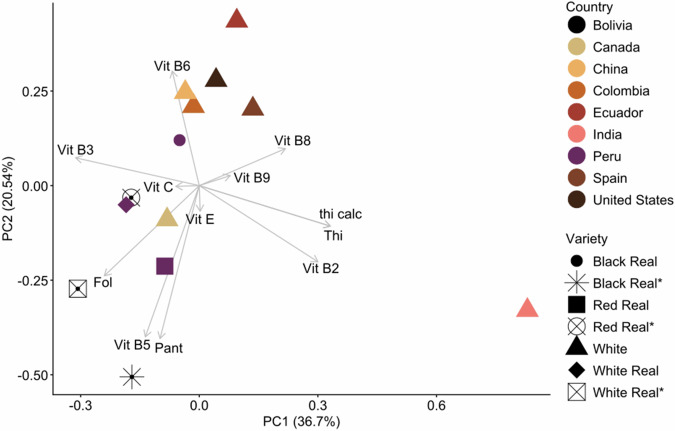
Principal Component Analysis (PCA) biplot portraying the quinoa samples and their corresponding fatty acid profiles. PC1 and PC2 explain 60% of the total variance. The biplot portrays the relationship between quinoa samples and their fatty acid composition. PC1 and PC2 explain 60% of the total variance. Arrows indicate the contribution of each fatty acid to the sample distribution.

PCA was used to identify distinctive patterns among the fatty acid profiles of the 13 samples (Fig. 7). The results show that Bolivian Quinoa Real differ from samples collected in other countries in certain properties. For example, all the Quinoa Real varieties from Bolivia are positioned at one end of the graph, characterised by oleic acid (especially red Real and white Real). In contrast, black Quinoa Real from Bolivia is characterised by its high palmitic acid content. The first principal component (PC1) accounted for 41.0% of the total variance. It represented a strong gradient driven by saturated fatty acids, particularly short- and medium-chain SFA such as C4:0, C6:0, C10:0, C14:0, and C15:0, all of which showed high negative loadings. The total SFA (TSFA) also contributed significantly to this axis, reinforcing the interpretation that PC1 captures overall variation in saturated lipid fractions.

In contrast, polyunsaturated fatty acids such as C18:2 and total PUFA exhibited positive loadings, indicating that PC1 primarily distinguishes lipid profiles rich in saturated fatty acids from those rich in polyunsaturated fatty acids. The second principal component (PC2), accounting for 19.3% of the variance, was linked to variation in specific long-chain MUFA and PUFA. Strong negative loadings were observed for C18:2 trans, C20:1, C20:2, C22:0, and C20:4n6, suggesting these fatty acids influence the negative end of this axis. Conversely, C12:0 and C18:3 showed the strongest positive contributions to PC2. This pattern indicates that PC2 captures a secondary gradient contrasting lipid profiles dominated by long-chain trans and n-6 fatty acids with those containing relatively higher amounts of certain medium-chain saturated fatty acids and n-3 polyunsaturated fatty acids.

**Fig. 8:**
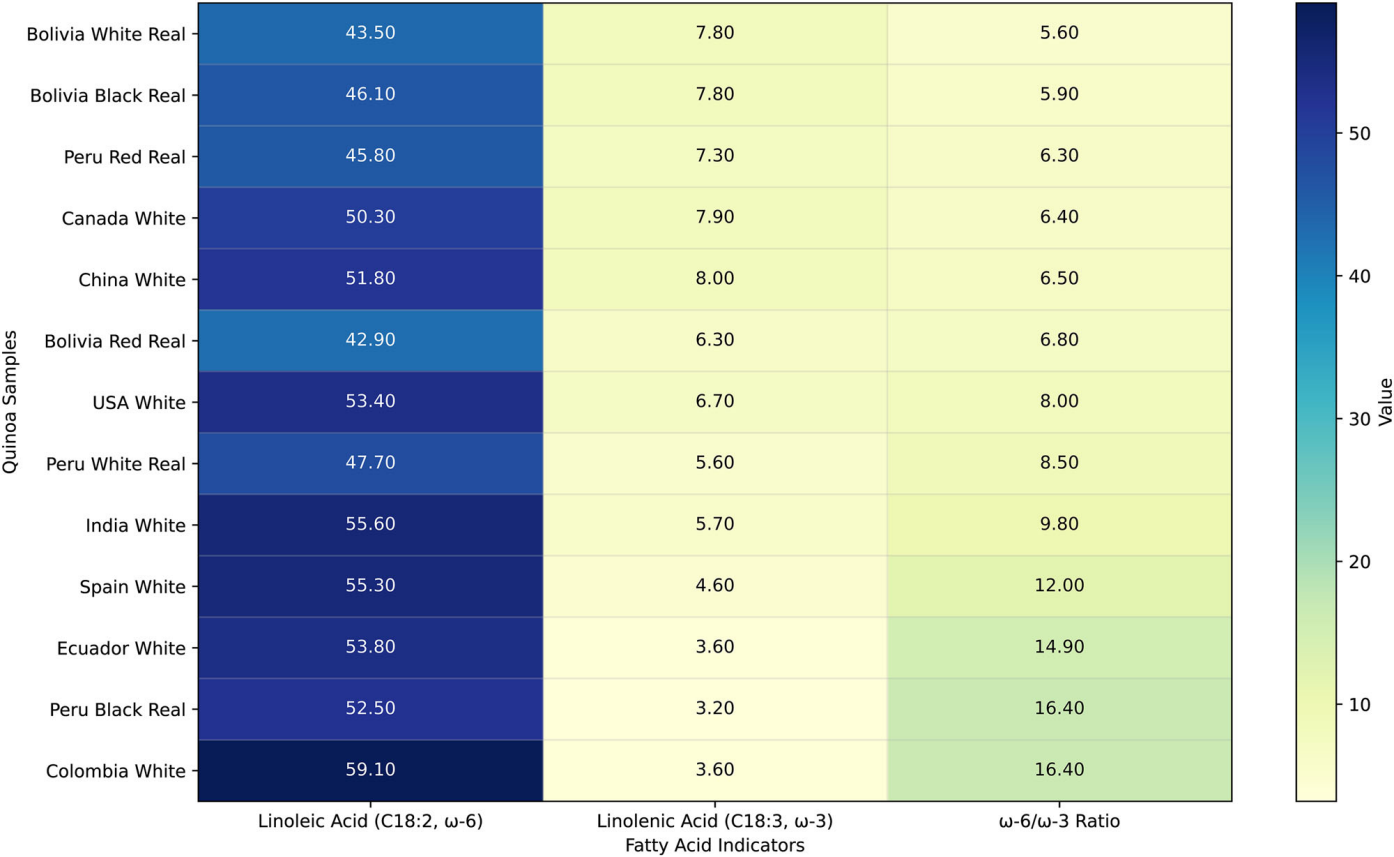
Essential fatty acids and ω−6/ω-3 ratio in quinoa samples. The samples are sorted by nutritional quality based on the ω-6/ω-3 ratio. Dark blue indicates a high concentration of fatty acid; light yellow indicates a low value. Lower ω-6/ω-3 is better for health. The values for linoleic and linolenic acids are in %.

The unique features of Quinoa Real varieties from Bolivia include higher levels of saturated fat ( ~ 16%), suitable shelf life, an excellent ω-6/ω-3 balance ( ~ 5.6–5.9%), and higher levels of monounsaturated fat. Interestingly, Bolivian quinoa real varieties, particularly Real white and black types, exhibit a unique lipid signature characterised by a superior ω6/ω-3 balance, elevated levels of heart-healthy monounsaturated fats, and reduced trans-fat content, suggesting nutritional superiority and enhanced oxidative stability compared to other global quinoa sources (Fig. 8).

### Vitamins

Quinoa is an essential source of vitamins^45,46^. In the present research, 15 vitamins were measured (Table 5). The findings are in accordance with the literature^35,45–47^. The results show significant levels of B6 and folate in Quinoa Real varieties from Bolivia, and of riboflavin, particularly in white quinoa from India, with Quinoa Real meeting more than 80% of the daily needs of children and more than 40% of those of adults^35^. There are high amounts of thiamine in white quinoa from India. The white quinoa from China and the three Quinoa Real varieties from Bolivia had higher levels of vitamin C than the other samples. These results suggest that Quinoa Real from Bolivia are essential source of vitamins B6, Folate, Riboflavin and Vitamin C for the human diet.

**Table 5:** Vitamins from 13 quinoas collected in different countries

Components	Unit	Bolivia			Peru			Ecuador	Colombia	USA	Canada	España	China	India
		White Real	Red Real	Black	White Real	Red Real	Black	White	White	White	White	White	White	White
Vitamin A (Retinol)	μg/100 g	< 21*	< 21*	< 21 *	< 21 *	< 21 *	< 21 *	< 21 *	< 21 *	< 21 *	< 21 *	< 21 *	< 21 *	< 21 *
Vitamin B3 (Total Niacin)	mg/100 g	0.743	0.684	0.696	0.74	0.817	0.713	0.733	0.624	0.56	0.819	0.552	0.806	0.151
Vitamin B8 – biotin	μg/100 g	15.1	16.7	19.9	17.1	20	19.2	30.2	13	19.6	24.6	26.7	22.1	27.8
Folate	μg/100 g	359	350	234	214	226	144	-	-	118	321	-	160	-
Vitamin B12	μg/100 g	< 0.25*	< 0.25*	< 0.25*	< 0.25*	< 0.25*	< 0.25*	< 0.25*	< 0.25 *	< 0.25*	< 0.25*	< 0.25*	< 0.25*	< 0.25*
Vitamin D3 (Cholcalciferol)	IU/kg	< 100*	< 100*	< 100*	< 100*	< 100*	< 100*	< 100*	< 100*	< 100*	< 100*	< 100*	< 100*	< 100*
Vitamin B2 – Riboflavin	mg/kg	0.356	0.508	0.556	0.436	0.626	0.498	0.497	0.439	0.409	0.451	0.398	0.491	1.19
Pantothenic acid (vitamin B5)	mg/kg	11.4	9.08	17.9	12.2	13.3	7.94	7.28	8.83	5.01	11	7.63	7.13	9.81
Pantothenic acid calc. As calcium salt	mg/kg	12.4	9.87	19.4	13.3	14.4	8.63	7.91	9.6	5.44	12	8.3	7.75	10.7
Thiamine (vitamin B1)	mg/kg	3.06	2.89	2.5	3.24	3.44	2.77	3.55	3.79	3.47	3.96	4.06	4.21	11.5
Thiamine. As thiamine chloride. hydrochloride	mg/kg	3.88	3.68	3.17	4.12	4.36	3.51	4.5	4.81	4.41	5.03	5.16	5.35	14.6
Vitamin C (Nutr. Suppl + premix)	mg/100 g	1.31	0.945	0.907	< 0.5	< 0.05 *	< 0.05 *	< 0.5	< 0.05 *	< 0.5	< 0.05 *	< 0.5	2.3	< 0.5
Vitamin B9. Total Folate	mg/100 g	-	-	2.34	-	2.26	1.44	2.2	1.73	1.18	2.26	1.3	1.6	1.37
Vitamin E (alpha-tocopheryl acetate)	mg/100 g	2.58	2.94	3.01	3.05	3.19	2.29	2.96	3.54	2.64	5.04	2.6	3.05	3.14
Vitamin B6-HCI	mg/100 g	0.213	0.212	0.115	0.294	0.198	0.169	0.38	0.331	0.183	0.242	0.173	0.297	0.144

*Below indicated quantification level.

**Fig. 9:**
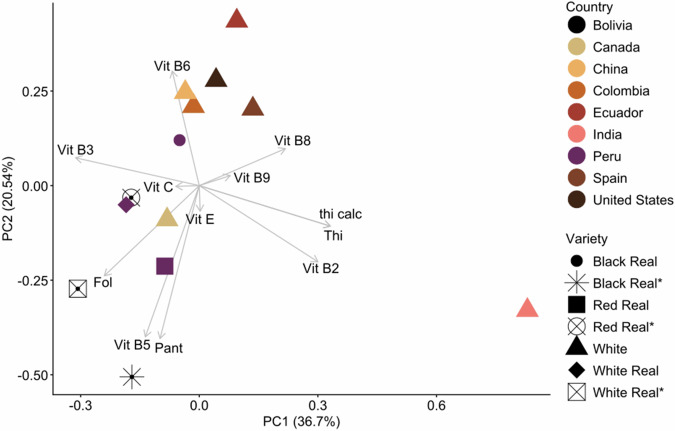
Principal Component Analysis (PCA) biplot portraying the quinoa samples and their corresponding vitamins. The biplot illustrates the relationship between quinoa samples and vitamin composition. Arrows represent the contribution of each vitamin to the variation among samples.

PCA was performed on the vitamin data set, which includes all samples (Fig. 9). The results showed that white and black Quinoa Real from Bolivia differ from the other samples in their folate, vitamin B5, and pantothenic acid content. Also, red Quinoa Real from Bolivia expresses vitamin B3 similarly to white Real from Peru. The samples from Ecuador, the USA, China, and Colombia, and white quinoa from Peru, are closely located and express vitamin B6. The quinoa samples from Spain and India are represented by vitamin B8, vitamin B2, and thiamine, respectively. White quinoa from India excels in B-complex vitamins, which are nutritionally interesting because they are involved in energy metabolism and neuronal function. Meanwhile, Quinoa Real from Bolivia is an essential source of vitamins B6, folate, riboflavin, pantothenic acid, and vitamin C for the human diet, particularly black quinoa.

PC1 (36.7%) was dominated by strong negative loadings for vitamin B and folates, along with notable negative contributions from vitamin B5, pantothenic acid, and vitamin C. In contrast, positive loadings for thiamine and calculated thiamine, vitamin B2, and vitamin B8 placed these B-complex vitamins at the opposite end of the axis. Therefore, PC1 reflects a trade-off between thiamine/riboflavin-rich profiles and varieties characterised by higher levels of niacin, folate, and pantothenic acid. Meanwhile, PC2 (20.5%) was mainly characterised by strong negative loadings for vitamin B5 and pantothenic acid, accompanied by moderately negative contributions from vitamin B2 and thiamine. Conversely, positive loadings were found for vitamin B6 and vitamin B3. These results suggest that PC2 represents a gradient contrasting B6-rich varieties with those exhibiting higher concentrations of pantothenic acid, B5, and riboflavin.

### Minerals and trace elements

The mineral and trace element content is shown in Table 6. A total of 18 parameters were determined in the samples. The values of the mineral composition are consistent with the literature-reported data^45^^,^^47^^–^^50^. Quinoa is also rich in micronutrients. For instance, Mg, Mn, Cu, and Fe meet daily nutritional needs, according to^35^. Regarding trace elements, the values were in accordance with the literature^45,49,50^. However, antimony (Sb) for quinoa was not found in the literature.

**Table 6:** Minerals and trace elements from quinoas collected in different countries

Components	Unit	Bolivia			Peru			Ecuador	Colombia	USA	Canada	Spain	China	India
		White Real	Red Real	Black Real	White Real	Red Real	Black	White	White	White	White	White	White	White
Aluminium (Al)	mg/kg	54	61	84	27	2.8	7.3	2.3	13	< 0.5	1.3	17	1.9	1.9
Antimony (Sb)	mg/kg	< 0.05	< 0.05	< 0.05	< 0.05	< 0.05	< 0.05	< 0.05	< 0.05	< 0.05	< 0.05	< 0.05	< 0.05	< 0.05
Arsenic (As)	mg/kg	< 0.1	< 0.1	< 0.1	< 0.1	< 0.1	< 0.1	< 0.1	< 0.1	< 0.1	< 0.1	< 0.1	< 0.1	< 0.1
Lead (Pb)	mg/kg	0.06	0.06	0.09	< 0.5	< 0.05	< 0.05	< 0.05	< 0.05	< 0.05	< 0.05	< 0.05	< 0.05	< 0.05
Boron (B)	mg/kg	8.1	12	20	9.8	6	8.1	9.1	5.4	9.1	8.3	9.3	7.9	10
Cadmium (Cd)	mg/kg	0.06	0.03	0.04	0.02	0.02	0.03	<0.01	0.02	0.03	0.07	0.04	0.02	0.04
Chromium (Cr)	mg/kg	0.33	0.23	1.8	0.12	0.33	0.31	0.13	0.12	0.08	< 0.05	0.35	0.12	0.07
Copper (Cu)	mg/kg	4.5	4.9	4.4	3.9	3.9	4.4	6.8	4.2	5.7	5.5	6.3	6.3	5.1
Nickel (Ni)	mg/kg	0.2	0.2	0.8	0.5	0.7	0.5	0.5	0.2	1.1	0.7	0.6	0.3	0.3
Silicon (Si)	mg/kg	120	130	200	61	8	21	7	12	2	4	35	5	9
Tin (Sn)	mg/kg	< 0.2	< 0.2	< 0.2	< 0.2	< 0.2	< 0.2	< 0.2	< 0.2	< 0.2	< 0.2	< 0.2	< 0.2	< 0.5
Lithium (Li)	mg/kg	< 0.5	< 0.5	< 0.5	< 0.5	< 0.5	< 0.5	< 0.5	< 0.5	< 0.5	< 0.5	< 0.5	< 0.5	< 0.5
Calcium (Ca)	mg/kg	490	510	480	540	280	560	500	470	310	330	540	370	390
Iron (Fe)	mg/kg	58	72	71	44	29	36	44	40	32	42	57	51	55
Potassium (K)	mg/kg	9600	12000	13000	8300	6000	7500	6500	7300	6900	7600	8200	5700	5400
Cobalt (Co)	mg/kg	< 0.1	< 0.1*	< 0.1*	0.1	0.1	< 0.1*	0.1	< 0.1*	< 0.1*	< 0.1*	< 0.1*	< 0.1*	< 0.1*
Magnesium (Mg)	mg/kg	1500	1500	1600	1800	1300	1700	1900	1700	1800	1800	1600	1800	1900
Manganese (Mn)	mg/kg	18	24	29	38	23	45	37	41	25	25	-	13	21
Sodium (Na)	g/100 g	0.008	0.005	0.007	0.002	< 0.001*	0.008	< 0.001*	0.003	< 0.001*	< 0.001*	0.005	0.001	0.011
Phosphorus (P)	mg/kg	3600	3600	3600	4000	3100	3700	4600	3600	4200	3100	-	4200	3800
Sulphur (S)	mg/kg	1300	1400	1300	1400	1300	1200	1400	1400	1500	1700	1600	1500	1700
Selenium (Se)	mg/kg	< 0.2*	< 0.2*	< 0.2*	< 0.2 *	< 0.2*	< 0.2*	< 0.2*	< 0.2*	< 0.2*	< 0.2*	< 0.2*	< 0.2*	0.2
Zinc (Zn)	mg/kg	30	25	26	28	23	27	33	29	28	24	27	22	24
Platinum (Pt)	mg/kg	< 0.05	< 0.05	< 0.05	-	< 0.05	< 0.05	< 0.05	< 0.05	< 0.05	< 0.05	< 0.05	< 0.05	-
Molybdenum (Mo)	mg/kg	0.2	0.4	0.3	0.2	0.1	0.2	0.2	0.2	0.2	0.1	0.3	0.2	0.1
Bismuth (Bi)	mg/kg	< 0.1*	< 0.1*	< 0.1	< 0.1*	< 0.1*	< 0.1 *	< 0.1*	< 0.1*	< 0.1*	< 0.1*	< 0.1	< 0.1 *	< 0.1

*Below indicated quantification level.

Bolivian Quinoa Real varieties also have the highest ash content, indicating high mineral content. Their values range from 2.9 to 3.6 g/100 g, while quinoa from other countries contains from 1.9 to 2.7 g/100 g (Table 2). Mainly, Bolivian black Real is unique in terms of essential minerals. It has the highest sum of Ca, Fe, K, Mg, P, and Zn, making it the most mineral-rich variety (Table 6). Both black and red Real varieties from Bolivia also stand out for the highest potassium levels, 13,000 and 12,000 mg/Kg, respectively. Potassium is essential for muscle function, neuronal signalling and heart health. Both varieties contain some of the highest Fe content, black at 72 mg/Kg and red at 71 mg/Kg. Iron is crucial for oxygen transport and for preventing anaemia. Bolivian Quinoa Real is rich in magnesium (1500–1600 mg/kg) and phosphorus (3600 mg/kg), contributing to bone health and metabolism. The Zn content is also noteworthy in Quinoa Real varieties from Bolivia, ranging from 20 to 30 mg/Kg. Zinc is essential for immune function and wound healing. Quinoa Real varieties from Bolivia, especially black Real, are richer in crucial minerals than quinoas from Peru, Ecuador, the USA, Canada, China, India, and Spain. This makes them nutritionally superior and potentially more beneficial for health.

**Fig. 10:**
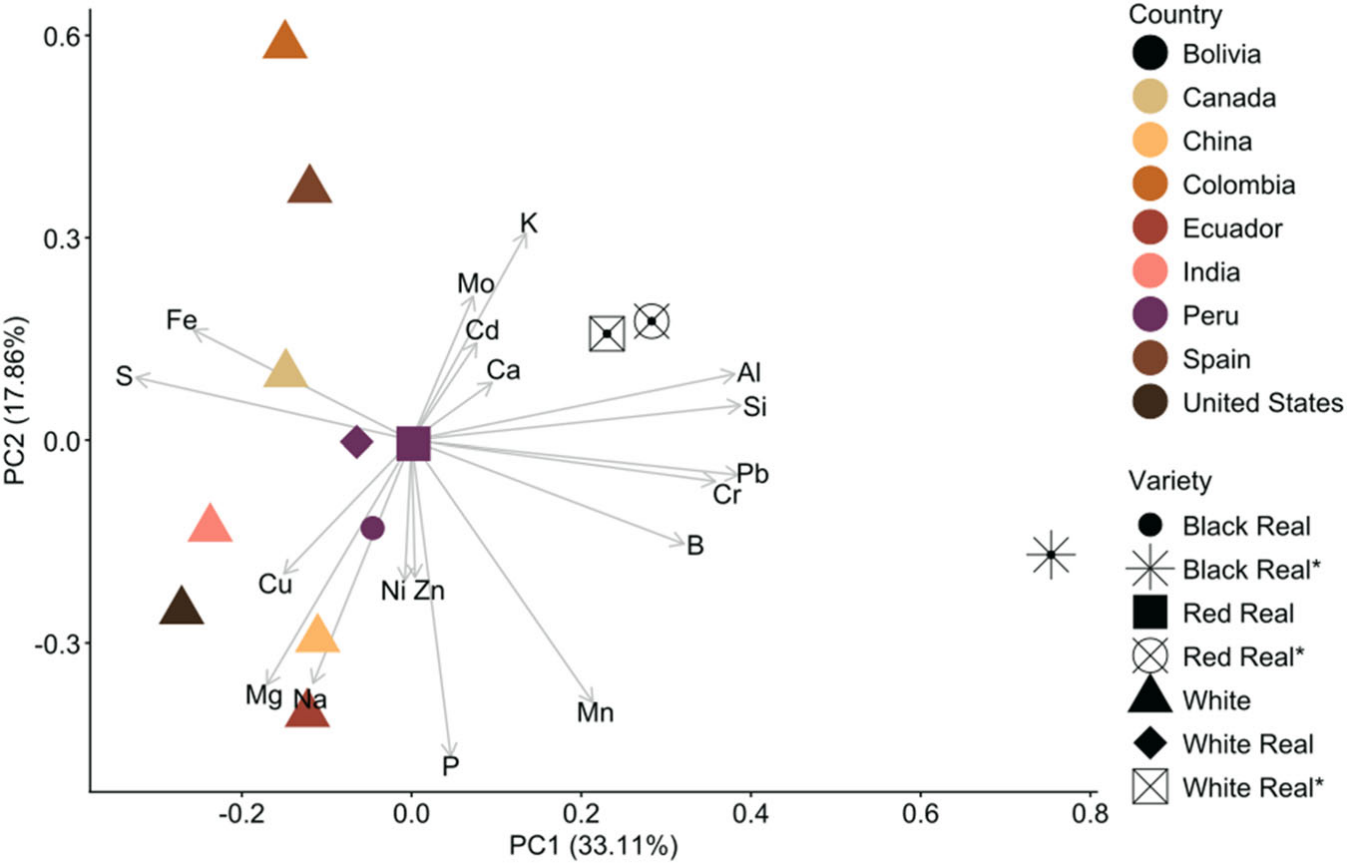
Principal Component Analysis (PCA) biplot portraying the quinoa samples and their corresponding minerals and trace elements. The biplot shows the relationship between quinoa samples and mineral/trace element composition. Arrows indicate the contribution of individual elements to the sample distribution.

Principal component analysis (PCA) was performed on the mineral data set, which includes all samples (Fig. 10). The Quinoa Real varieties from Bolivia are separated from those from other regions. For example, the Quinoa Real samples from Bolivia contain Al, Si, Ca, Cd, Mo, and K. In contrast, the quinoa from Ecuador, China, India, and the USA is characterised by Na, Mg, Cu, Zn, Ni, and P, while Fe dominates the white quinoa from Canada. Iron and K dominate the samples from Spain and Colombia.

Regarding the Peru sample, it was in the centre of the graphic. PC1 and PC2 explain 51% of the total variance. These results show that differences among samples can be due to soil composition^51^ and indicated a particular characteristic of the region from which the samples were collected. Therefore, it can be considered a factor in chemical differentiation.

The PC1, which explained 33.1% of the total variance, was characterised by strong positive loadings of Al, Pb, B, Cr, and Si. This pattern indicated that PC1 primarily reflects variation in lithogenic or soil-derived elements, suggesting that differences among samples could be driven by edaphic conditions or soil-plant transfer of geogenic minerals. Moderate positive contributions from Ca and Mn reinforce this interpretation, as both elements often co-vary with geochemical signatures in the substrate. In contrast, essential nutrients such as Fe, Mg, Na, and S loaded negatively on PC1, indicating that samples enriched in lithogenic elements tended to show lower concentrations of physiologically regulated minerals. Thus, PC1 delineates a clear gradient separating samples influenced by environmental or substrate-derived mineral inputs from those characterised by higher levels of metabolically controlled nutrients. The PC2, accounting for 17.9% of the variance, captured a different dimension of variability linked to plant nutrient physiology. Strong negative loadings for Mg, Na, Mn, and P, together with moderate negative contributions from B and Zn, point to coordinated variation among elements associated with metabolic functions, osmotic regulation, and enzymatic activity. Conversely, K, Mo, Fe, and Cd exhibited positive loadings on PC2, revealing an opposing pattern related to nutrient uptake dynamics or stress-mediated element accumulation. The contrasting behavior of K and Mg, both major nutrients, suggests differences in nutrient availability or in the plant’s ion homeostasis across samples. Overall, PC2 reflects a nutrient-balancing gradient that is independent of the lithogenic signature captured by PC1.

### Non-metric multidimensional scaling (NMDS)

To visualise and explore the similarities and differences between the quinoa samples across all parameters that differed, we performed non-metric multidimensional scaling (nMDS), an unconstrained ordination technique, using the metaMDS function in the vegan package in R^52,53^. We aimed to evaluate compositional patterns in quinoa using Bray-Curtis similarity distance. Then, we used the envfit function in the vegan package in R to fit the parameter vectors to the nMDS ordination^52^.

**Fig. 11:**
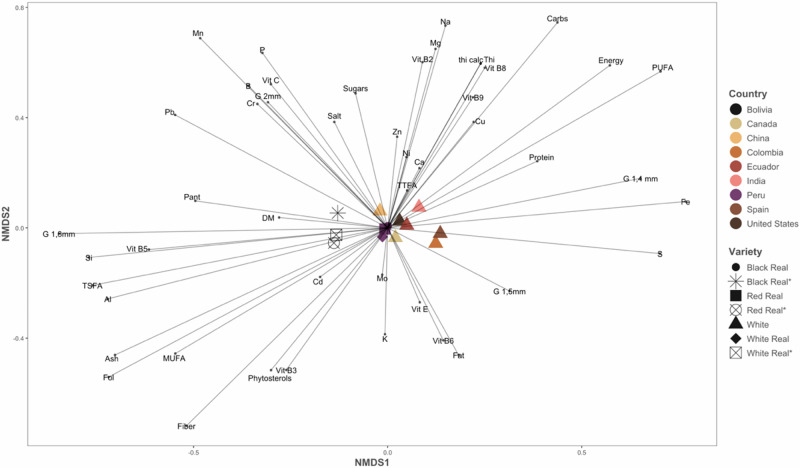
Non-metric multidimensional scaling (nMDS) ordination of the chemical composition of quinoa. Each point on the ordination is a different quinoa. The shape of each point represents the quinoa type, and the points are coloured by origin country.

The results are presented in Fig. 11, representing Quinoa Real varieties from Bolivia. The results of the nMDS analysis revealed a clear separation of Bolivian Quinoa Real varieties from the rest, forming a distinct cluster on nMDS 1 (stress: 0.08). In addition, the ordination shows their content on monounsaturated fatty acids (MUFA), particularly oleic acid, grain size (1.8 mm), some minerals and trace elements (Al, B, Mn, P, Pb, Si, etc.), as well as vitamins such as B5, pantothenic acid, vitamin C, etc. It is also clear that Quinoa Real from Bolivia differs in its fibre, phytosterol, and amino acid profiles.

On the opposite end, samples from Colombia, Spain, India, and Ecuador are characterised by the content of polyunsaturated fatty acids (PUFA), some minerals and trace elements (S, Ni, Ca, etc.), as well as vitamins such as B6, thiamine, B8, etc., and protein and carbohydrate content. Finally, samples from Peru, Canada, and China are located close to the centre of the graphic, indicating that these samples have similar compositional patterns or are more similar to each other than to other samples.

## Discussion

The chemical composition of quinoa samples collected from various countries generally aligns with previous findings^4^^,^^39^^,^^48^. However, the extreme climatic and edaphic conditions are expected to significantly affect plant metabolism and, consequently, contribute to the unique composition observed in Quinoa Real grains. Saline and N-deficient soils could be the main factors in the mineral and protein composition of Quinoa Real from Bolivia. The higher ash content and elevated levels of K, Mg, Fe, and Zn, especially in black Quinoa Real from Bolivia, are consistent with enhanced ion uptake and storage under saline and mineral-rich conditions. On the other hand, the N limitation may constrain the total protein content, explaining the comparatively lower protein levels in some Quinoa Real samples. At the same time, long-term selection under stress may favour genotypes that allocate N efficiently into high-quality storage proteins and essential amino acids. This interpretation is supported by proteomic work showing that salinity can strongly remodel quinoa seed storage proteins, particularly 11S globulins (chenopodins), and increase the accumulation of free essential amino acids in the salt-tolerant Salares ecotype of Chilean quinoa landraces^54^.

The intense UV radiation at high altitudes can induce oxidative stress, prompting the biosynthesis of antioxidant compounds. The higher levels of vitamin C, folate, pantothenic acid, some B-vitamins, and phytosterols in Quinoa Real from Bolivia are compatible with such a stress-induced enhancement of antioxidant and protective metabolism. In addition, salinity studies in quinoa similarly report increased phenolic content and antioxidant activity in seeds exposed to high NaCl concentrations, particularly in stress-tolerant genotypes from Chilean quinoa landraces^54^^.^

Several experimental studies on quinoa cultivated across diverse agroecological conditions offer a framework for understanding current results on genotype × environment interactions. For example, a study involving three quinoa cultivars (Regalona, Salcedo-INIA, and Titicaca) grown in Spain, Chile, and Peru reported that several components, including amino acid profiles, protein content, mineral composition, and phytate content, vary by location and cultivar. On the other hand, fibre content and saponin amounts remained relatively stable across different locations^55^. Our results support these findings, indicating that many nutritional attributes in quinoa vary with agroecological conditions, while a smaller set of traits show greater stability. For instance, the higher fibre and phytosterol contents observed in Quinoa Real from Bolivia compared with other commercial samples suggest that this ecotype may be beyond the range of variability documented in previous multi-environment trials, emphasising its importance as a distinct nutritional resource.

Another study investigated the impact of heat stress on quinoa seed oil content and fatty acid composition in southern Spain^41^. Five new varieties adapted to European conditions (Pasto, Marisma, Jesie, Roja, and Duquesa) were analysed. Over two years, episodes of heat stress decreased oil content, lowered MUFA levels, increased PUFA levels, reduced the oleic acid/linoleic acid ratio, and raised the ω-6/ω-3 ratio to about 9:1. Thus, these changes diminished the quinoa’s nutritional value. Conversely, Quinoa Real from Bolivia demonstrated a more favourable ω-6/ω-3 ratio (5.6–5.9:1), high levels of monounsaturated fatty acids, and low trans-fat content, particularly in the white and black cultivars. These differences are linked to the generally cooler climate at the high altitudes of the *Intersalar* zone and large diurnal thermal amplitudes rather than prolonged heat episodes.

Heat stress has also been shown to influence the mineral composition of quinoa seeds. Following heat treatment, the main factor affecting the seed’s elemental composition was the timing of panicle development relative to the heat exposure period^56^. An 11-day heat treatment during anthesis of the main panicle caused significant changes in the elemental profiles of 20 elements. Therefore, the mineral composition is highly sensitive not only to soil chemistry and genotype but also to short-term thermal events and phenological stages.

The present study provides a robust comparative picture of the nutritional profile of Bolivian Quinoa Real by analysing a wide set of commercial samples from different regions in a single accredited laboratory using harmonised analytical protocols. Inevitably, working with market-available products implies some uncertainty regarding upstream post-harvest handling, storage and processing. However, because all samples were treated identically upon arrival and intra-sample variation was carefully controlled, any residual processing effects are expected to be secondary to the major differences observed among origins and ecotypes. Thus, while such factors cannot be completely excluded, they are unlikely to account for the consistent clustering of Quinoa Real and its markedly higher levels of fibre, phytosterols, vitamins and minerals.

The mechanisms underlying this nutritional diversity, including the relative contributions of genetic background and edaphoclimatic factors, remain incompletely understood. The present findings therefore provide a rationale for targeted experimental studies in which Bolivian Quinoa Real varieties are grown under controlled abiotic conditions (e.g., salinity, temperature, water availability) alongside representative non-Real cultivars. Such common-garden and multi-stress experiments would make it possible to disentangle genetic and environmental effects on the accumulation of nutrients and functional components, and to identify the key physiological and molecular determinants of the nutrient-dense phenotype of Quinoa Real.

The principal distinguishing features of Bolivian Quinoa Real, relative to quinoa cultivated in other countries, include its higher levels of dietary fibre, ash, phytosterols, and essential minerals, particularly K, Fe, and Mg. It also exhibits a more favourable fatty-acid profile, characterised by elevated oleic acid and a health-promoting omega-6 to omega-3 ratio, as well as higher levels of several vitamins, notably vitamin C, folate, pantothenic acid, and riboflavin, together with a well-balanced essential amino acid profile. In addition, Quinoa Real is characterised by its larger grain size, which contributes to its distinctive morphology.

Quinoa Real explicitly refers to the quinoa cultivars grown exclusively in the *Intersalar* zone of southern Bolivia, located between the Uyuni and Coipasa salt flats, at altitudes ranging from 3,800 to 4,800 m.a.s.l. These highland ecosystems are characterised by extreme aridity, high UV radiation, and large daily temperature fluctuations, which contribute to the unique composition of quinoa grains. Traditional Andean farming communities have preserved and selected these cultivars over generations, giving rise to the cultivars now recognised as Quinoa Real.

Finally, these findings support the hypothesis that Bolivian Quinoa Real, an ancient crop, is a unique nutritional resource with nutritional and functional superiority. Bolivian Quinoa Real is an attractive candidate for health-oriented markets and sustainable agricultural practices.

## Methods

The chemical composition of Quinoa Real was compared with that of other commercial varieties produced in various geographical areas worldwide, including Peru, Ecuador, Colombia, the USA, Canada, Spain, China, and India. The samples were obtained from regions where quinoa cultivation is prevalent, covering both traditional and non-traditional areas, ensuring a comprehensive and reliable comparison. Thirteen samples were analysed using international standardised methods to determine their chemical components of nutritional relevance, including protein, amino acids, fatty acids, carbohydrates, vitamins (A, B1, B2, B3, B5, B6, B12, C, and D3), minerals, and trace elements. Additionally, granulometry was determined to explore potential morphological differences.

### Sample collection and laboratory analysis procedures

Thirteen quinoa seed samples were collected from nine countries between March and April 2021 (Table 7). All samples are commercially available products from established companies, including Andean Valley Company (Bolivia), INIA and Live Kinoa (Peru), Cereales Andinos (Ecuador), Karavansay (Colombia), Lundberg Family Farms (USA), NorQuin (Canada), Algosur (Spain), Kilaru Naturals PVT (India), and Jiaqui Quinoa (China). Because these were commercial products subjected to standardised industrial processing and quality control, the samples were considered homogeneous within each batch. Therefore, a single sample from each company was deemed representative of the corresponding product.

**Table 7:** Origin and source of the Quinoa samples studied

Country	Company/Source	Type
Bolivia	Andean Valley Company	White Real*
		Red Real*
		Black Real*
Peru	INIA and Live Kinoa	White Real
		Red Real
		Black
Ecuador	Cereales Andinos	White
Colombia	Karavansay	White
USA	Lundberg Family Farms	White
Canada	Nor Quin	White
Spain	Algosur	White
India	Kilaru Naturals PVT	White
China	Jiaqui Quinoa	White

*Royal Quinoa.

All samples were processed and analysed by Eurofins (Germany), an internationally accredited laboratory, using validated and certified methods in accordance with ISO/IEC 17025:2017. This ensures traceability, precision, and compliance with internationally recognised quality-assurance procedures. Although the analytical report did not include replicate-level data, Eurofins´ accreditation guarantees the use of internal replications, calibration, and control of analytical methods.

### Granulometry

The granulometry was determined using sieves with openings of 1.4 mm, 1.5 mm, 1.8 mm, and 2.0 mm, according to the method NF ISO 2591-1:1998 and following the grain size standard for quinoa established by the Codex Alimentarius Commission (2020)^33^. The results were expressed as percentages (ISO 2591-1:1998).

### Proximate Composition Analysis

Protein content (N × 6.25) was determined using the Kjeldahl digestion technique, followed by volumetric analysis of the ammonia formed. Fat content was measured by exhaustive extraction in a Soxhlet apparatus using petroleum ether. Ash content was determined based on the inorganic residue remaining after combustion. Moisture content was determined using the oven-drying method, in which the sample was dried at 135°C for 2 hours, following the *Lebensmittel- und Futtermittelgesetzbuch* [LFGB] (2020) § 64 *Official Collection of Test Procedures* (LFGB § 64), issued by the German Federal Ministry of Food and Agriculture. The total carbohydrate content was calculated as the difference between 100 and the sum of moisture, protein, total lipids, total fibre, and ash. All measurements were performed according to the reference methods specified in § 64 LFGB

### Amino acids

The amino acid profile was determined using ion chromatography coupled with UV detection, according to the method EU 152/2009 (F) and ISO 13903:2005, except for tryptophan, which was determined by liquid chromatography with fluorescence detection (LC-FLD). The results were expressed as g/100 g of sample (EU 152/2009 (F); ISO 13903:2005).

### Fatty acids

Before fatty acid analysis, the samples were esterified and then measured by gas chromatography with flame ionisation detection (GC-FID) according to the method: DGF C-VI 11 d, 1998 SOP1209-04 (Deutsche Gesellschaft für Fettwissenschaft, 2017). The results were expressed as percentages.

### Sugars

The sugar spectrum was determined by high-performance anion-exchange chromatography with pulsed amperometry detection (HPAE-PAD), a widely used technique for determining carbohydrates^57^. The results were expressed as g/100 g (sugar/sample).

### Vitamins

Vitamin A was determined by liquid chromatography with diode array detection (LC-DAD) according to EN 12823-1 2014. The results were expressed as µg/100 g of sample.

Vitamin B1 (HCl) (thiamine hydrochloride) was determined by liquid chromatography with fluorescence detection (LC-FLD) according to method EN 14122-2014. The results were expressed as mg/100 g of sample.

Vitamin B2 - Riboflavin was determined by liquid chromatography with fluorescence detection (LC-FLD) according to method EN 14152:2014 mod. Results were expressed as mg/kg samples.

Vitamin B3 (total niacin) EN-HPLC was determined by liquid chromatography with fluorescence detection according to method EN 15652:2009. Results were expressed as mg/100 g sample. (LC-FLD).

Vitamin B5 LC/MS (mg/kg) (pantothenic acid), calculated as calcium pantothenate, was determined by liquid chromatography-mass spectrometry (LC-MS) according to method AOAC 2012.16.^58^. Results were expressed as mg/kg of sample.

Vitamin B8 – Biotin microbiological, using a method that involves leveraging the growth response of specific microorganisms that require biotin for their proliferation, determined by nephelometry according to FDA method LST AB 266.1,1995, 866. Results were expressed as µg/100 g of sample.

Folate microbiological, using a method that involves leveraging the growth response of specific microorganisms that require folate for their proliferation, determined by nephelometry according to the technique NMKL 111:1985, 870. The results were expressed in µg/100 g of sample.

Vitamin B12 HPLC (Immuno) Food & Feed was determined by liquid chromatography with ultraviolet and diode array detector (LC-UV/DAD) according to the method J. AOAC 2008, vol 91 No 4^59^ The results were expressed as µg/100 g of sample.

Vitamin C (Nutr. Suppl + premix) was determined by liquid chromatography-mass spectrometry (LC-MS) according to ISO 20635:2018. Results were expressed as mg/100 g of sample.

Vitamin E (α-tocopheryl acetate) was determined by liquid chromatography with fluorescence detection (LC-FLD) according to EN 12822:2000. Results were expressed as mg/100 g of sample.

Vitamin B6-HCl) was determined by liquid chromatography with fluorescence detection (LC-FLD) according to method EN 14164:2014. Results were expressed as mg/100 g sample.

Vitamin D3 (cholecalciferol) was determined by liquid chromatography with a diode array detector. Vitamin B12 HPLC (Immuno) Food & Feed was determined by liquid chromatography with ultraviolet and diode array detector (LC-UV/DAD) according to the method J. AOAC 2008, vol 91 No 4^59^. The results were expressed as µg/100 g of sample.

Vitamin D3 (cholecalciferol) was determined by liquid chromatography with diode array detection (LC-DAD) according to method EN 12821:2009. Results were expressed as IU/kg sample.

### Minerals and trace elements

Antimony (Sb), arsenic (As), bismuth (Bi), cadmium (Cd), chromium (Cr), cobalt (Co), lead (Pb), molybdenum (Mo), nickel (Ni), selenium (Se) and tin (Sn) were determined by inductively coupled plasma mass spectrometry (ICP-MS) according to the method DIN EN ISO 17294-2 (2017-01), mod., CON-PV 01274 (2017-12) following the DIN EN ISO 17294-2:2017-01.

Aluminum (Al), boron (B), calcium (Ca), copper (Cu), iron (Fe), lithium (Li), magnesium (Mg), manganese (Mn), phosphorus (P), potassium (K), silicon (Si), and sulfur (S) were determined by inductively coupled plasma optical emission spectroscopy (ICP-OES) according to DIN EN ISO 11885 (2009-09), mod. CON-PV 00006 (2020-01) following the DIN EN ISO 11885.

Sodium (Na) was determined by flame atomic absorption spectroscopy (F-AAS) and platinum by ICP-MS according to DS/EN 13805:2014 and DS/EN ISO 17294 m:2016. The results were expressed in mg/kg of sample, except for phosphorus, which was expressed as mg/100 g.

### Data Analysis and Statistical Methods

The dataset was analysed using Microsoft Excel 2016 (Microsoft, Redmond, WA, USA), and the results were expressed as mean values of six replicates. All measurements were below the 5% coefficient of variation, and the limits of detection and quantification were determined. Heatmaps were generated in Python 3, and figures were created in the vector graphics editor *Inkscape v1.0*.

Principal component analysis (PCA) and non-metric multidimensional scaling (nMDS), an unconstrained method, were carried out using the statistical ordination technique language R version 4.2.3 (R Foundation for Statistical Computing, Vienna, Austria).

## Data Availability

Raw data will be made available on request to the corresponding authors.
